# Environmental Sampling Using Shoe Booties for the Detection of Foot and Mouth Disease Virus in Animal Pens

**DOI:** 10.3390/v18070759

**Published:** 2026-07-10

**Authors:** Judy Hodge, Sean Yeo, Kate Hole, Hanh H. T. Nguyen, Wei Shern Lee, Jack S. Richards, Sonja Laurendeau, Charles Nfon, Shawn Babiuk

**Affiliations:** 1 National Centre for Foreign Animal Diseases, Canadian Food Inspection Agency, 1015 Arlington Street, Winnipeg, MB R3E 3R2, Canada; judy.hodge@inspection.gc.ca (J.H.); sean.yeo@inspection.gc.ca (S.Y.); kate.hole@inspection.gc.ca (K.H.); charles.nfon@inspection.gc.ca (C.N.); 2 ZiP Diagnostics Pty Ltd., Collingwood, VIC 3066, Australia; hanh.n@zipdiag.com (H.H.T.N.); wei.l@zipdiag.com (W.S.L.); jack.r@zipdiag.com (J.S.R.); 3 Ruminant and Swine Programs, Animal Health Directorate, Canadian Food Inspection Agency, 1081 Main Street, Moncton, NB E1C 8R2, Canada; sonja.laurendeau@inspection.gc.ca

**Keywords:** foot and mouth disease, FMDV, early detection, shoe bootie, environmental sampling, oral fluids, outbreak response

## Abstract

The consequences of foot and mouth disease (FMD) outbreaks in non-endemic countries are devastating. Early detection and confirmation of foot and mouth disease virus (FMDV) are critical for controlling outbreaks. Diagnostic sampling currently uses direct sampling of suspected animals, including swabs and/or vesicular fluid. In the event of an FMD outbreak, there is limited veterinary capacity to sample all animals in the control zones. Thus, environmental sampling methods (shoe booties, ropes, and environmental swabs) were assessed and compared to direct animal sampling for experimentally infected cattle and swine. Environmental sampling methods such as shoe booties were sufficient to identify FMDV by reverse transcription quantitative PCR (RT-qPCR) and nanopore sequencing, similarly to oral swabs collected from animals. Furthermore, a loop-mediated isothermal amplification (LAMP)-based point-of-care test produced by ZiP Diagnostics was able to rapidly detect FMDV from clinical and environmental samples with Cq values of 30 or less. This study demonstrates a proof of concept that shoe booties can be used to detect FMDV in animal pens prior to the onset of clinical signs in animals. The use of shoe booties for surveillance sampling of non-clinical animals on multiple premises in a given zone would increase operational capacity and enable earlier detection for a faster response to FMD outbreaks.

## 1. Introduction

Foot and mouth disease (FMD) is caused by FMD virus (FMDV), which belongs to the *Picornaviridae* family under the *Aphthovirus* genus. It infects key livestock species including sheep, goats, cattle, swine, and other cloven-hoofed animals [1]. FMDV has seven serotypes: O, A, C, Asia-1, and Southern African Territories (SAT)-1, SAT-2, and SAT-3 [2]. Serotype C has not been detected since 2004 [3]. FDMV is endemic in seven geographic pools with an uneven distribution of serotypes in Asia, the Middle East, Africa, and parts of South America [4]. There have been recent outbreaks of FMDV occurring in Germany [5], Slovakia, and Hungary [6]. The impact of these outbreaks was the countries lost their status of being FMD-free without vaccination with the World Organization of Animal Health (WOAH), resulting in the countries being barred from exporting animals or animal products, with subsequent economic losses [7].

Early detection and rapid diagnosis of FMDV is critical in mitigating the damage from an outbreak and regaining disease-free status. Following the index case of an FMDV outbreak in Canada, the Canadian Food Inspection Agency (CFIA) will lead the foreign animal disease emergency response. Different control zones would be established, including an infected zone and a restricted zone. In control zones, sampling of non-clinical animals to collect blood samples and nasal swabs is performed. Samples are assessed for the presence of FMDV genome using reverse transcription quantitative PCR (RT-qPCR) and nanopore sequencing via the FMDV-ONTAPS protocol. In addition, serology can be used with a non-structural 3ABC protein (NSP) ELISA to demonstrate freedom of disease in both vaccinated and unvaccinated animals [8].

Pigs and cattle infected with FMDV can shed the virus, which is capable of infecting other susceptible animals prior to displaying clinical signs of disease [9,10]. The level of viral shedding and transmission differs among animal species. FMDV shedding in swine is known to be higher compared to cattle and FMDV transmission from infected pigs to cattle or goats occurs more easily than in the opposite way [11,12].

Since FMDV can be detected prior to animals displaying clinical signs of disease, active surveillance can be used to identify if proximity and high-risk contact premises are infected before animals show clinical signs. Individual animal sampling can be performed for active surveillance, but this is labor-intensive and costly. Alternative sampling methods have been identified including oral fluids [13]; however, oral fluid collection is only suitable for use in swine, as cattle will not chew ropes for oral fluid collection. Environmental sampling methods, such as swabs of flooring or water and food troughs [14] and air sampling [15], are an attractive option for simplicity and speed. Shoe booties are an additional environmental sampling method which has demonstrated effectiveness for *Mycobacterium avium* subspecies *paratuberculosis* (MAP) detection in dairy cows [16]. The advantage of shoe booties compared to other environmental sampling methods is that these samples can be collected without the need for veterinarians trained in foreign animal disease inspection. Shoe booties can simply be placed over footwear worn by farmers while inspecting their animals for common clinical signs of FMDV. The shoe booties can subsequently be submitted to veterinary authorities for laboratory analysis. The objective of this study was to compare environmental samples to conventional oral swabs, and to assess whether shoe booties can be used to identify infected animal pens at a rate comparable to those of traditional direct animal sampling methods.

## 2. Materials and Methods

### 2.1. Sample Acquisition and Preparation

All clinical samples were obtained from an animal experiment approved by the National Centre for Foreign Animal Disease Animal Care Committee, animal use document number C-22-008. The animals included 2 Holstein calves at 6 months of age and 4 White-cross pigs at 4 weeks of age. The calves were housed together in one cubicle, while the pigs were housed together in another cubicle.

The animals were infected with FMDV O UKG 11/2001 (received from The Pirbright Institute, Ash Road, Pirbright, Surrey, England) that was propagated in primary lamb kidney cells. The animals were inoculated intranasally at a dose of 10^7^ TCID_50_ per animal. The calves and two pigs were inoculated intranasally on the first day, while the other two pigs were inoculated the following day, both intranasally and via heel-bulb injection. The samples were collected from all animals over a period of four days. The samples collected included shoe booties, water dish swabs, food dish swabs, toy swabs (pigs only), oral fluids, and nasal swabs of animal care staff entering the animal cubicles. Toys are provided in the pig cubicles for enrichment, but they are not used with cattle, so two different water dishes were sampled in the cattle cubicles instead. Traditional samples such as oral swabs, lesion swabs, vesicular fluid, and epithelial tissues were also collected. To collect the shoe bootie samples, animal care staff donned disposable, absorbent shoe covers (ULINE, Milton, ON, Canada) over their regular steel-toed rubber boots (gumboots/Wellies) (ULINE) and walked around in the animal cubicles for at least one minute, repeated with a total of 4 sets of booties per cubicle per day. The booties were then removed, dirty side out (containing absorbed feces, urine and water), and placed into resealable Ziplock plastic bags to which 10 mL of sterile Dulbecco’s Phosphate Buffered Saline (D-PBS) was added, with focus placed on the bottom of the bootie. The booties with D-PBS were squeezed to help extract and collect as much material as possible, with the liquid being retained as the shoe bootie sample.

Oral swabs were collected from each animal and stored in 1 mL of Universal Viral Transport Media (UTM) (Becton, Dickinson, and Company, Mississauga, ON, Canada). Environmental swabs were taken in each cubicle from food dishes, water dishes, and toys and placed into tubes containing in 1 mL UTM. As part of the environmental sampling, animal care staff had nasal swabs taken when exiting the cubicles. These were also stored in 1 mL of UTM. All swabs were vortexed to release the virus prior to RNA extraction. Ropes (TEGO™ Swine Oral Fluids Kit, ITL BioMedical, Melbourne, Australia) were hung in the pig cubicle for a minimum of 10 min. The ropes were then placed in a plastic bag, squeezed to extract the saliva as per the manufacturer’s instructions, and the saliva was transferred to a clean tube. On the last day of the experiment, the pigs were reluctant to chew on the rope, so an additional 10 mL buffer was added to the ropes. Oral fluids were clarified by centrifugation at 2000× *g* for 20 min at 4 °C prior to RNA extraction. Epithelial tags were collected post-mortem and placed in UTM for storage. For testing, 10% weight/volume tissue suspensions were made in D-PBS. These suspensions were also clarified by centrifugation (2000× *g* for 20 min at 4 °C) prior to RNA extraction.

### 2.2. RNA Extraction and Real-Time RT-PCR

RNA extraction on all sample types was performed using the Applied Biosystems MagMax-96 Viral RNA Isolation Kit (ThermoFisher Scientific, Burlington, ON, Canada), following the manufacturer’s protocol. RNA was extracted using a KingFisher APEX (ThermoFisher Scientific) and then assessed for FMDV positivity by RT-qPCR, as described previously [17]. Briefly, 5 μL of template RNA was added to a 20 μL mastermix, comprising TaqMan^®^ Fast Virus 1-Step master mix (ThermoFisher Scientific), 0.5 μM of each of the forward and reverse primers, and 0.2 μM of the probe. RT-qPCR was performed on a QuantStudio™ 7 Pro (ThermoFisher Scientific) machine using the following cycling conditions: 50 °C for 5 min, 95 °C for 20 s (s), followed by 40 cycles of 95 °C for 15 s and 60 °C for 45 s. A cut-off quantification cycle value (Cq) of ≤35.99 was considered to be positive for the presence of the FMDV genome in the sample [17].

### 2.3. ZiP-FMD-P2 Point-of-Care Testing

A subset of environmental samples (46 of 92) was tested via the ZiP-FMD-P2 platform (ZiP Diagnostics, Collingwood, Victoria, Australia) following the manufacturer’s instructions with modifications. The ZiP-FMD-P2 kits utilize a LAMP assay targeting the 3D gene that differs from existing RT-qPCR assays. Briefly, this platform is designed for use in point-of-care settings where animal swabs are immediately added to buffer tube 1 to start the assay process. For our purposes, as the samples needed to be tested with multiple assays, 100 µL of sample was added to buffer tube 1 instead. After this point, the machine prompts were followed as per manufacturer instructions.

### 2.4. Isolation and DAS-ELISA

Isolation was performed on the 46 samples tested by RT-qPCR and the ZiP-FMD-P2 platform. Briefly, monolayers of a porcine kidney cell line expressing α_v_β_6_ integrin (LFBK α_v_β_6_), which were previously misidentified as a calf kidney cell line [18], were inoculated and the plates incubated for 2–3 days at 37 °C with daily monitoring for CPE. The LFBK αvβ6 cell line is a sensitive cell line for isolating FMDV. Negative samples underwent a second cell culture passage. All positive samples were confirmed to be FMDV by RT-qPCR and serotype O by DAS-ELISA. The classical DAS-ELISA for FMDV serotype O was performed as described previously [19].

### 2.5. Nanopore Sequencing

Nanopore sequencing was performed as previously described [20]. Briefly, cDNA template was produced from extracted RNA using SuperScript™ IV First-Strand Synthesis System (ThermoFisher Scientific) and a universal FMDV poly (A) primer. FMDV P1 PCR amplicons were generated from the cDNA template with the Platinum™ SuperFi II PCR Master Mix (ThermoFisher Scientific) and pan-serotype FMDV primers targeting the P1 region. Amplicons were confirmed with gel electrophoresis first and followed by two cleanup steps with AMPure XP magnetic beads (Beckman Coulter, Mississauga, ON, Canada). Cleaned amplicons were barcoded with the Rapid Barcoding Kit (ONT, Oxford, UK) using a modified protocol and sequenced with Flongle Flow Cells (ONT) on a MinION Mk1D [20].

Raw sequencing data was processed as done previously [20]. Briefly, reads were mapped using Minimap2 (v2.28) [21] against a custom database of reference FMDV sequences. Mapping statistics and consensus sequences were generated using Samtools (v1.21) [22].

## 3. Results

### 3.1. Progression of Clinical Signs in Swine and Cattle Following Experimental Infection with FMDV

Cattle and swine were experimentally infected with FMDV and evaluated for clinical signs of disease. Fever was detected in both cattle and swine starting at DPI2 prior to the presence of vesicles observed on DPI3 and DPI4 at the end of the study. On DPI2 in the morning, both calves were bright, alert, and responsive with no evidence of salivation or lameness, although calf #59 had a fever. In the afternoon of DPI2, calf #59 was lame, with a ruptured vesicle on one foot, and was given an anti-inflammatory (ketoprofen). On DPI3, both calves had fevers, drooling, lameness, and vesicles were present in the mouth, on all 4 feet, and on the teats. Both calves were given an anti-inflammatory on DPI3 and euthanized on DPI4. On DPI1 and 2, all 4 pigs were bright, alert, and responsive with no signs of illness. On DPI3, the pigs had eaten all their food overnight, but were all depressed and reluctant to move, with Pigs #17 and #20 having vesicles present on their feet and Pig #19 was frothing at the mouth. All pigs were given an anti-inflammatory (meloxicam) and an opioid (buprenorphine) for pain control. At the afternoon check, Pigs #18 and #19 had severe lesions on their feet and were euthanized. At the night check, the remaining 2 pigs were depressed, reluctant to move, and not eating. They both received another dose of buprenorphine and were euthanized the next morning (DPI4).

### 3.2. Detection of FMDV Genome in Samples Collected from Animals and the Environment

**Figure 1:**
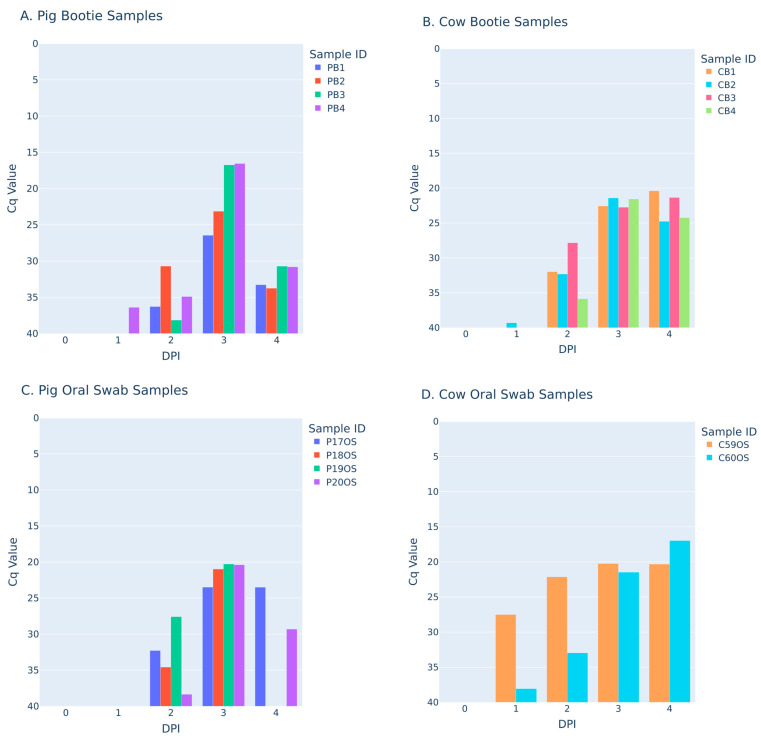
Comparison of Cq values from RT-qPCR from shoe bootie environmental samples and oral swab sampling. DPI: days post-infection. (**A**) PB1–4 represents different animal care staff booties worn in the pig cubicle. (**B**) CB1–4 represents different animal care staff booties worn in the cow cubicle. (**C**) P17OS–P20OS are individual pig oral swab samples. (**D**) C59OS and C60OS are individual cow oral swab samples.

The main purpose of this study was to determine whether environmental sampling, such as shoe booties, could be as effective as traditional sampling by oral swabs to identify infected premises. To prove this, all shoe bootie and oral swab samples were initially tested by RT-qPCR and the results compared (Figure 1).

**Figure 2:**
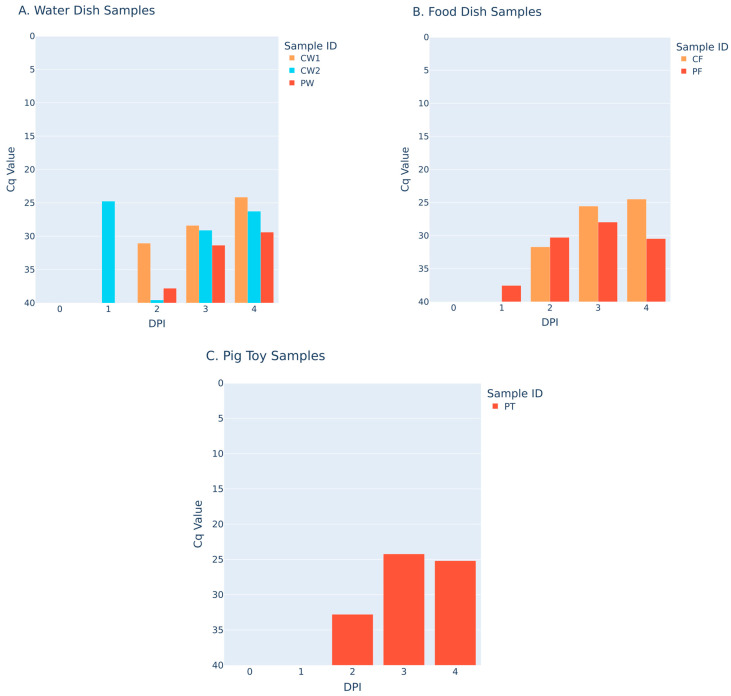
Comparison of other environmental samples by RT-qPCR. (**A**) CW refers to the water dishes in the cow cubicle of which there are two, PW refers to the water dish in the pig cubicle. (**B**) CF and PF refer to the cow food dish and the pig food dish in their respective cubicles. (**C**) PT refers to pig toys.

Both shoe botties and oral swabs when tested gave positive results using RT-qPCR by DPI2, an increase in quantity of viral genome was detected at DPI3, and sample types were still positive at DPI4, albeit with a decreased level of viral genome being detected. Similarly, other environmental samples (dishes, toys) were positive using RT-qPCR by DPI2 and had an increased level of viral genome present by DPI3 and DPI4 (Figure 2).

### 3.3. Evaluation of the ZiP-FMD-P2 Point-of-Care System

A subset of environmental samples (46 of 92) was tested using the ZiP-FMD-P2 point-of-care system which is based on a LAMP assay platform. Of these 46 samples, 5 were negative and 41 were positive by FMD RT-qPCR. These environmental samples included shoe booties, food/water dishes, toy, and oral fluids. Human nasal swabs were also obtained from animal care staff to determine whether this sample type could be used as an indication of disease presence. A viral dilution series was tested on the ZiP-FMDV-P2 assay to determine the limitations and its potential use in the field. The limit of detection (LoD) testing demonstrated that the ZiP assay is approximately one log less sensitive than RT-qPCR since the limit of detection for the 10^−7^ dilution is right at the limit of detection (Cq 35.99) (Table 1).

Of the 41 PCR positive samples, 20 were also positive on the ZiP assay (Table 2). In general, samples with Cq values greater than 30 trended negatively on the ZiP assay. A Cq of around 30 equates to approximately 10^2^ TCID_50_ for the RT-qPCR assay. A + represents as positive test result and a − represents a negative test result.

The samples evaluated by the RT-qPCR and ZiP-FMDV-P2 assay were evaluated for viable virus using virus isolation. Many of the samples when placed on cells caused cytopathic effect (CPE) observed in the cells, indicating the presence of a live virus. To confirm the observed CPE in isolation was directly caused by FMDV, positive isolates were assessed by both RT-qPCR and DAS-ELISA specific for serotype O. All cells with CPE observed were positive by both RT-qPCR and DAS-ELISA. From that, it can be concluded that FMDV replication took place resulting in CPE which was observed and recorded. A + represents as positive test result and a − represents a negative test result.

### 3.4. Evaluation of Oxford Nanopore Technologies Amplicon P1 Sequencing Protocol on a Subset of Samples

**Figure 3:**
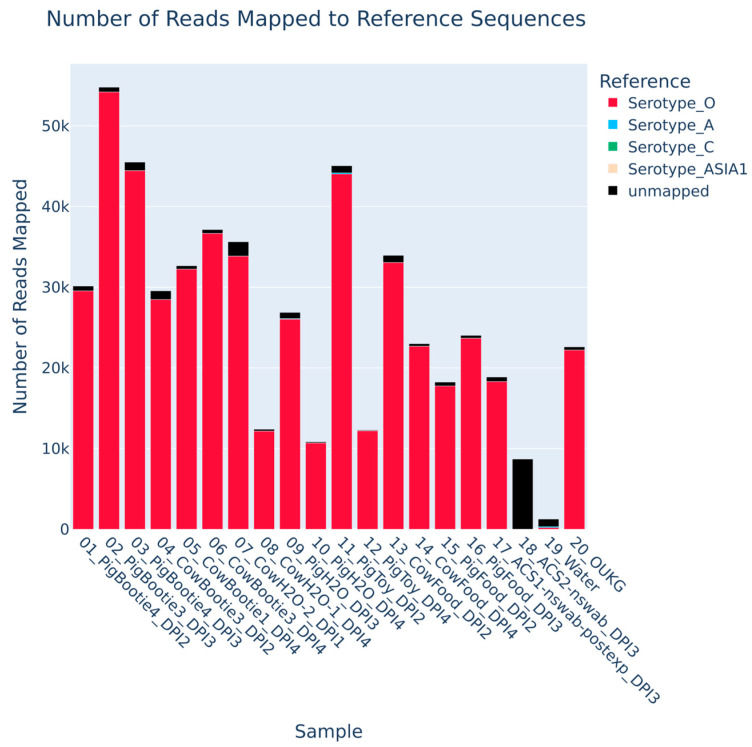
Nanopore sequencing of select environmental samples. Data shows the number of reads mapped to FMDV O reference sequences (red color). Reads that did not map to any FMDV reference sequence are shown in black.

The robustness of P1 nanopore sequencing from environmental samples was evaluated to determine whether they could also be used to quickly identify FMDV in these same environmental samples, and whether they contained anything inhibitory that would impact the assay. The results of the Nanopore sequencing demonstrated that environmental samples with a Cq range between 16.55 and 34.90 were successfully sequenced (Figure 3, Table 2).

Sequence coverage was >99% for all samples at a read depth of 50× or 1% of the mean read depth—whichever is higher (Table 3). The inverse unmapped to total read ratio indicates that all selected samples tested with the FMDV Oxford Nanopore Technologies Amplicon P1 Sequencing (FMDV-ONTAPS) method succeeded. All samples had ratios >0.97 except for the ACS2 nasal swab sample.

## 4. Discussion

Early detection of FMDV is critical to control FMD. This is illustrated from the recent FMDV outbreaks in Europe, where the outbreaks were rapidly identified and controlled [5,6]. Germany employed complete stamping-out while Hungary and Slovakia chose stamping-out combined with emergency vaccination. FMDV can be detected in animals prior to the onset of vesicles [9]. However, there is a lag in positive FMDV diagnosis with passive surveillance due to testing being requested only when vesicular lesions are observed on susceptible animals. Various sample matrices have been used to detect FMDV including but not limited to oral and nasal swabs, oral fluids [23], meat juice [24], and various environmental samples from both experimental [14,15] and field situations [25,26,27]. The purpose of this study was to compare environmental to a conventional animal sampling method of oral swab collection and to other sampling methods to determine whether these samples would be suitable for surveillance. In addition, evaluation of the point-of-care ZiP Diagnostics assay for FMDV detection was also tested to determine useability of environmental samples with a point-of-care test.

This study demonstrated that various environmental sample matrices such as oral fluids via ropes, water bowl swabs, feed bowl swabs, toy swabs, and shoe bootie samples were useful for detecting FMDV (Figure 1 and Figure 2). The shoe bootie sampling method has the advantage of being easy to perform by farmers, animal handlers, or veterinarians without difficulties, simply by placing the shoe booties over their rubber boots. This is especially important during FMDV outbreaks as there are capacity limitations of veterinary services for the collection of traditional animal samples.

RT-qPCR is the primary diagnostic assay used to detect FMDV due to its high sensitivity, superb specificity, and rapid turnaround time of 3–4 h [17]. However, performance of this test is limited to laboratories with specialized equipment and highly trained analysists—making this diagnostic assay difficult to implement in the field as a point-of-care test. Alternatives such as LAMP isothermal assays can be performed easily in the field compared to RT-qPCR [28]. The ZiP-FMD-P2 kit is one such LAMP isothermal assay that targets the 3D gene of FMDV—the same target used in existing RT-qPCR assays (Callahan—Pirbright/USDA) for FMDV detection [29]. ZiP can detect the specific FMDV O serotype at around 10^2^ TCID50/mL or 1 log less than the diagnostic RT-qPCR (Table 1). In this study, the ZiP-FMD-P2 LAMP assay was evaluated as a point-of-care diagnostic test. By combining shoe bootie sampling with the ZiP Diagnostics point-of-care test, sample collection and diagnostic testing for FMDV detection is possible without expertise in conducting complex laboratory assays. This is important for rapid identification of FMDV in endemic regions lacking veterinary and diagnostic services, or during outbreaks in either endemic or non-endemic countries. The ZiP Diagnostics point-of-care test can provide results in as little as 10 to 20 min at the cost of reduced sensitivity. The quick turnaround time and diminished sensitivity is due to the lack of a robust sample RNA extraction step. RNA extraction introduces a major complication for point-of-care diagnostics—it requires multiple types of equipment and various reagent storage temperatures. Hence, this step is omitted in the ZiP test workflow for easy point-of-care testing, despite the lower sensitivity of the test.

The shoe bootie samples could contain inhibitors that potentially reduce detection in both the RT-qPCR and ZiP Diagnostics LAMP assay. However, the environmental sample types tested give acceptable Cq values similar to conventional sample types currently used to assess farm status. This is evident with the Cq values of the shoe bootie and oral swab samples (Figure 1). Furthermore, the ZiP assay was capable of detecting FMDV containing samples at the limit of detection determined by using virus in the absence of inhibitors (Table 1).

Nanopore sequencing of select environmental samples was successfully performed utilizing the FMDV-ONTAPS protocol (Figure 3, Table 3). The almost perfect coverage across all selected samples demonstrates recovery of the FMDV O UKG 11/01 virus used in this study for animal inoculation (Table 3). P1 sequence data produced from the select environmental samples was comparable, and in some cases exceeds what was observed previously with traditional samples [20]. Previously, the sensitivity of the FMDV-ONTAPS protocol was limited to samples with a RT-qPCR Cq value of approximately 28 or less [20]. In this case, samples with Cq values up to 34.90 were sequenced successfully. This highlights the importance of fresh samples and quick processing to ensure the greatest assay sensitivity with the FMDV-ONTAPS protocol. The one exception is a single sample (ACS2 Nswab DPI 3) with a Cq value of 31.35 that could not be sequenced (Table 2). It is well known that FMDV can be transmitted easily between farms simply from contaminated matter travelling on footwear of individuals [30]. Therefore, it is possible that this sample did not produce meaningful sequencing results due to the lack of organic matter to protect the low levels of virus in the sample from RNA degradation. In contrast, shoe bootie samples contained high amounts of organic matter which may have contributed to viral longevity. FMDV half-life based on sample matrix could be investigated further in the future with more concrete studies. Additionally, samples with higher Cq values reflect the low copy number of the viral genome. In these cases, the likelihood of pipetting copies of the viral genome for sequencing is significantly decreased and leads to unreliable PCR amplification. These results demonstrate the lack of inhibitors in assessed environmental samples that could interfere with sequencing, and that the FMDV-ONTAPS protocol is a reliable method for detection of FMDV.

Confirmation of the molecular diagnostics using virus isolation demonstrated the presence of live FMDV in many samples which were positive by RT-qPCR with Cq values between 16–36. Success with this assay could be attributed to the experimental design. The experiment was conducted on site, and samples were processed shortly after collection. In this case, long-distance transportation was not a factor. Under normal circumstances, samples must travel far which can affect virus viability; thus, the results from the virus isolation may not be as successful with field samples.

Furthermore, nasal swabs collected from animal care staff tested positive for the presence of FDMV by both RT-qPCR and virus isolation (Table 2), indicating that FMDV can be inhaled by people in the presence of FMDV-infected animals. This agrees with a previous study demonstrating that FMDV can be detected by RT-qPCR in nasal swabs collected from humans [31].

The study was performed using both swine and cattle, which did not show major differences in the kinetics or magnitude of FMDV secretions based on results from the RT-qPCR, LAMP assay (ZiP), virus isolation, and P1 sequencing with the FMDV-ONTAPS protocol. Environmental sampling for FMDV has been explored in a goat market in Nepal, demonstrating the effectiveness of this approach [25]. Additional studies using sheep and goats in an experimental setting would further support the utility of shoe booties to detect FMDV.

This study was conducted in CL3 Ag high containment facilities which are under negative pressure and have high levels of air exchange to remove airborne FMDV. Despite this, shoe booties were still effective as a fomite for detecting FMDV. Further studies are required to determine the effectiveness of shoe bootie sampling in the field. Swine and dairy barns would act as similar environments as high containment animal cubicles with respect to solid flooring and walls. Ranches and feedlots differ in having grassland to be sampled; however, the virus can be transported between farms through contaminated boots [30]. Therefore, it is likely this sampling method would be useful to use in the field situation for ranches.

One of the study limitations was the sampling, which was performed using multiple infected animals in a confined space. Further evaluation of the shoe bootie sampling method using a single infected animal within a similar space would be important to further demonstrate the effectiveness of this sampling method. In addition, shoe booties could be used to detect additional vesicular diseases such as Senecavirus A, swine vesicular disease and vesicular stomatitis; however, this requires further testing.

## 5. Conclusions

This study demonstrates that shoe bootie samples are an effective sample matrix, comparable to oral swabs, for the detection of FMDV with RT-qPCR diagnostics. Shoe bootie sampling is a simple, noninvasive sampling method that can easily be performed by farmers. Further studies evaluating shoe bootie sampling in field conditions are required before this sampling method can be implemented for active surveillance following an FMDV outbreak.

## Figures and Tables

**Table 1:** O UKG 11/2001 limit of detection determination. RT-qPCR Cq values less than 35.99 are positive.

Dilution	RT-qPCR Cq	ZiP-FMD-P2
10^−1^	15.86	+
10^−2^	18.80	+
10^−3^	22.12	+
10^−4^	25.36	+
10^−5^	28.95	+
10^−6^	31.96	−
10^−7^	35.64	−

**Table 2:** RT-qPCR, LAMP assay, virus isolation, and nanopore sequencing of various environmental samples.

Sample	DPI	TCID50	Cq	ZiP-FMDV-P2	Isolation	# Reads Mapped to FMDV O UKG 11/01
Pig Bootie 4	3	6.00	16.55	positive	+	44,464
Pig Bootie 3	3	5.94	16.75	positive	+	54,209
Pig Oral Fluids	3	5.77	17.30	positive	+	ND
Cow Bootie 1	4	4.84	20.39	positive	+	32,245
Cow Bootie 3	4	4.55	21.34	positive	+	36,684
Cow Bootie 2	3	4.52	21.42	positive	+	ND
Cow Bootie 1	3	4.18	22.56	positive	+	ND
Pig Toy	3	3.68	24.23	positive	+	ND
Pig Oral Fluids	2	3.62	24.43	positive	+	ND
Cow Food Dish	4	3.60	24.49	positive	+	22,674
Cow Water 2	1	3.51	24.77	positive	+	33,865
Cow Bootie 2	4	3.51	24.77	positive	+	ND
Pig Toy	4	3.39	25.19	positive	+	12,204
Cow Food Dish	3	3.28	25.55	positive	+	ND
Cow Water 2	4	3.06	26.27	positive	+	ND
Pig Bootie 1	3	3.00	26.45	positive	−	ND
Cow Bootie 3	2	2.59	27.83	positive	+	28,481
Pig Food Dish	3	2.54	27.97	positive	+	23,685
Cow Water 1	3	2.42	28.39	positive	+	12,178
Pig Water	4	2.11	29.40	negative	+	10,702
Pig Food Dish	2	1.85	30.28	positive	+	17,765
Pig Food Dish	4	1.79	30.47	negative	+	ND
ACS1 NSwab PostExp	3	1.77	30.54	negative	+	18,313
Pig Bootie 2	2	1.71	30.70	negative	−	ND
Pig Bootie 3	4	1.71	30.72	negative	−	ND
Cow Water 1	2	1.61	31.05	negative	−	ND
ACS2 Nswab	3	1.52	31.35	negative	+*	9
Pig Water	3	1.52	31.36	negative	+	26,042
Pig Oral Fluids	4	1.50	31.43	negative	−	ND
Cow Food Dish	2	1.41	31.71	negative	+	33,073
ACS3 Nswab	3	1.40	31.75	negative	+	ND
Cow Bootie 1	2	1.33	31.98	negative	+	ND
ACS1 Nswab	2	1.24	32.29	negative	+	ND
Cow Bootie 2	2	1.23	32.32	negative	+	ND
ACS2 Nswab	4	1.11	32.70	negative	−	ND
Pig Toy	2	1.09	32.79	negative	+	44,049
Pig Bootie 1	4	0.94	33.27	negative	−	ND
Pig Bootie 2	4	0.80	33.75	negative	−	ND
ACS4 Nswab	4	0.60	34.41	negative	−	ND
Pig Bootie 4	2	0.50	34.90	negative	−	29,542
ACS1 Nswab PostShower	3	0.17	35.81	negative	+	ND

Note: +*: second passage required to obtain isolate; ND: not done; ACS: animal care staff.

**Table 3:** Mean read depth, minimum read depth, coverage, and the inverse unmapped to total read ratio from nanopore sequencing of select various experimental samples. Minimum read depth is set to 50× or 1% of the mean read depth—whichever is higher.

Sample	Mean Read Depth	Min. Read Depth	Coverage	Inv. Unmapped to Total Read Ratio
PigBootie4_DPI2	10,712.69	107.13	99.24	0.9802
PigBootie3_DPI3	20,630.24	206.30	99.27	0.9892
PigBootie4_DPI3	16,993.33	169.93	99.27	0.9771
CowBootie3_DPI2	9639.78	96.40	99.24	0.9637
CowBootie1_DPI4	12,392.64	123.93	99.27	0.9873
CowBootie3_DPI4	13,207.47	132.07	99.27	0.9875
CowWater2_DPI1	12,975.94	129.76	99.27	0.9504
CowWater1_DPI4	4429.99	50.00	99.27	0.9822
PigWater_DPI3	10,204.91	102.05	99.27	0.9717
PigWater_DPI4	4050.90	50.00	99.27	0.9880
PigToy_DPI2	16,822.69	168.23	99.27	0.9804
PigToy_DPI4	4205.80	50.00	99.27	0.9928
CowFood_DPI2	13,158.38	131.58	99.27	0.9741
CowFood_DPI4	8758.24	87.58	99.27	0.9862
PigFood_DPI2	6846.57	68.47	99.27	0.9737
PigFood_DPI3	9170.06	91.70	99.27	0.9857
ACS1-nswab-postexp_DPI3	7406.41	74.06	99.27	0.9707
ACS2-nswab_DPI3	9.68	50.00	0.00	0.0010
Water	44.29	50.00	31.79	0.2589
FMDV O UKG 11/01	8553.92	85.54	99.27	0.9831

ACS: animal care staff.

## Data Availability

The original contributions presented in this study are included in the article.
